# UV-Vis Spectrophotometry and Multivariate Calibration Method for Simultaneous Determination of Theophylline, Montelukast and Loratadine in Tablet Preparations and Spiked Human Plasma

**Published:** 2016

**Authors:** Seyed Karim Hassaninejad-Darzi, Abdolraouf Samadi-Maybodi, Seyed Mohsen Nikou

**Affiliations:** a *Department of Chemistry, Faculty of Science, Babol University of Technology, Babol, Iran.*; b *Analytical Division, Faculty of Chemistry, University of Mazandaran, Babolsar, Iran.*

**Keywords:** UV–Vis spectrophotometry, Multivariate calibration 1, Theophylline, Montelukast, Loratadine

## Abstract

Resolution of binary mixtures of theophylline (THEO), montelukast (MKST) and loratadine (LORA) with minimum sample pre-treatment and without analyte separation has been successfully achieved by multivariate spectrophotometric calibration, together with partial least-squares (PLS-1), principal component regression (PCR) and hybrid linear analysis (HLA). Data of analysis were obtained from UV–Vis spectra of three compounds. The method of central composite design was used in the ranges of 2–14 and 3–11 mg L^–1^ for calibration and validation sets, respectively. The models refinement procedure and their validation were performed by cross-validation. The minimum root mean square error of prediction (RMSEP) was 0.173 mg L^−1^ for THEO with PCR, 0.187 mg L^–1^ for MKST with PLS1 and 0.251 mg L^–1^ for LORA with HLA techniques. The limit of detection was obtained 0.03, 0.05 and 0.05 mg L^−1^ by PCR model for THEO, MKST and LORA, respectively. The procedure was successfully applied for simultaneous determination of the above compounds in pharmaceutical tablets and human plasma. Notwithstanding the spectral overlapping among three drugs, as well as the intrinsic variability of the latter in unknown samples, the recoveries are excellent.

## Introduction

Asthma is a common chronic inflammatory disease of the airways characterized by variable and recurring symptoms, reversible airflow obstruction and bronchospasm. Common symptoms include wheezing, coughing, chest tightness, and shortness of breath. The prevalence of asthma has increased significantly since the 1970s. In 2011, 235–300 million people globally have been diagnosed with asthma, and it caused 250,000 deaths. The control of asthma symptoms is a realistic goal and studies have shown that this can be achieved in most asthma patients leading to a higher quality of life. In spite of this, the control of asthma is generally poor (1).

Theophylline (THEO) is one of the most commonly used medications for the treatment of the symptoms of chronic asthma. Physiological conditions such as heart failure, liver disease, infection and obesity are known to reduce THEO elimination. Also, this drug has a shortened half-life in smokers and in children (2). THEO has been determined in pharmaceutical preparations by several methods such as HPLC (3, 4), HPTLC (5), micellar electrokinetic chromatography (6), capillary electrophoresis (7), ion chromatography (8), spectrophotometry (9–11) and electrospray ionisation ion mobility spectrometry (12), fluorescence (13) and electrochemistry (14–16). Also, three chemometric–spectroscopic methods were reported for determination of THEO in blood serum (17) and syrups (18). Montelukast (MKST) is a potent and selective antagonist of the cysteinyl leukotriene receptor utilized for the treatment of asthma. Reviewing the literature revealed that MKST has been determined using several techniques such as HPLC (19–25), HPTLC (26, 27), electrochemistry (28) and spectrophotometry (29). Loratadine (LORA) is a long-acting tricyclic antihistamine with selective peripheral histamine H1-receptor antagonist activity that applied to treat allergies. LORA has been determined using several techniques like HPLC (30–33), HPTLC (34), gas–liquid–chromatography (35), GC-MS (36), capillary electrophoresis (37), electrochemistry (38, 39) and spectrophotometry (40–42).

The conventional spectrophotometric methods use a separate number of wavelengths that are not enough to offer frequently the necessary information to resolve a system with severe spectra overlapping (43–45). Several approaches have been proposed for elaboration of spectrophotometric data to extract analytical information from unresolved band spectra. In recent years, a number of reports were published by scientists about multicomponent analysis of complex drug mixtures (46–50). Multivariate methods allow extracting analytical information and permit a rapid analytical response with minimum sample preparation, reasonable accuracy and precision without separation procedures. For these reasons, these methods can be considered for routine analysis of the drugs in their pharmaceutical formulations.

Among different regression method existed for multivariate calibration, the factor analysis based methods including principal component regression (PCR) and partial least squares (PLS) have received considerable attention in the literature (51-54). An excellent review of the multivariate statistical method has been presented by Martens and Naes (52). Because PLS and PCR is a full-spectrum method, efficient outlier detection methods are available from spectral residuals, and limited chemically interpretable spectral information can be obtained from PLS in some cases. This advantage allows for a rapid determination of mixture components; often with no need of prior separation or sample pre-treatment (55). Hybrid linear analysis (HLA) can be applied when data for pure considered analyte is available (56). This ‘hybrid’ method combines the advantage of knowing pure component spectra (like classical least squares) with the modeling advantage of ignoring all other species (e.g., PLS and PCR). The main idea of HLA is to obtain a limited number of factors of a data matrix in which the contribution of the analyte of interest has been removed, and is therefore based on net analyte signal (NAS) calculation. Sensitivity, selectivity and the value of signal to noise ratio are among the valuable analytical information that can be achieved from NAS (57).

In respect of literature survey, no published method were employed for the simultaneously determination of theophylline, montelukast and loratadine that does not require a prior physical separation. In this study, a fast method is described for the determination of these drugs. This simultaneously determination method is based on the coupling of UV–Vis spectroscopy and chemometric multivariate calibration techniques, mainly PLS1, PCR and HLA.

## Experimental

*Materials*

**Figure 1:**
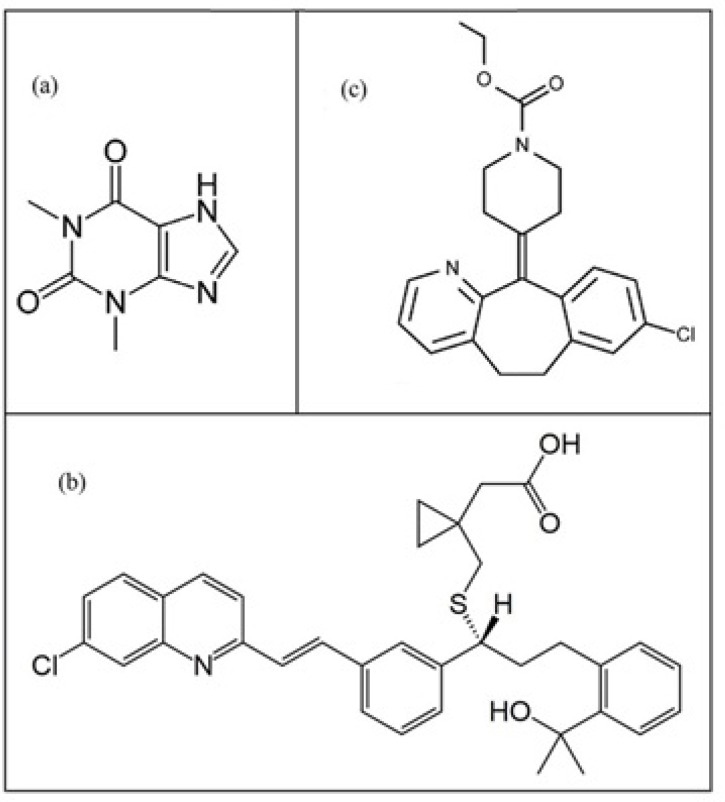
Molecular structure of three compounds in methanol: (a) theophylline, (b) montelukast and (c) loratadine.

Theophylline, montelukast and loratadine were purchased from Sigma-Aldrich and methanol was prepared from Fluka Company. All stock solutions were prepared by dissolving 20 mg of the corresponding compounds in 10 mL of methanol. The concentrations of THEO, MKST and LORA were 1.110×10^−2^, 3.411×10^−3^ and 5.223×10^−3^ mol L^-1^, respectively. Figure 1. displays the molecular structures of three drugs.

*Apparatus and software*

Absorption measurements were done using UV–Vis spectrophotometer (PG Instrument Ltd. – Model T90+). All measurements were carried out at 22 ^o^C using a quartz cuvette of 1.0 cm optical path. Data were handled using MATLAB software (version 7.8). PLS1, PCR and HLA were applied with programs written by Goicoechea *et al.* (55).

*Procedures*

*One component calibration*

In order to find the linear dynamic range (LDR) for each drug, different volumes of stock solution of each drug was added to 10 mL volumetric flask and diluted to the mark with methanol. The electronic absorption spectra were recorded over the range of 190–400 nm. Absorption values were measured at the wavelength of 275, 276 and 251 nm with different concentration of the THEO, MKST and LORA, respectively. The LDR for each compound was achieved by plotting absorbance versus drug concentration and were obtained 1.0–25.0 mg L^−1^ for all drugs.

*Calibration and validation sets*

The calibration set was built according to a central composite design (CCD). This design is construed as, three factors at two-level in the cubic vertex, six experiment in the cubic face and one central point (2^3^ + (2×3)+1). Concentrations of three drugs for the calibration set are presented in Table 1. that is in the known linear absorbance–concentration range of each drug. The concentration range of 2.0–14.0 mg L^−1^ was selected for each compound. The concentrations of three materials in prediction set that applied for validation of calibration model were chosen according to the central composite design except concentration range of three drugs was 3.0–11.0 mg L^−1^. Standard solutions of calibration and validation sets were prepared in 10 mL volumetric flasks by addition of appropriate amounts of each stock solution and diluted by methanol to the mark. UV–Vis spectra of the corresponding solutions were recorded in the same spectral conditions at ambient temperature (ca. 22 ^o^C). Data of the UV–Vis spectra were used for multivariate calibration 1 (MVC1) analysis.

**Figure 2:**
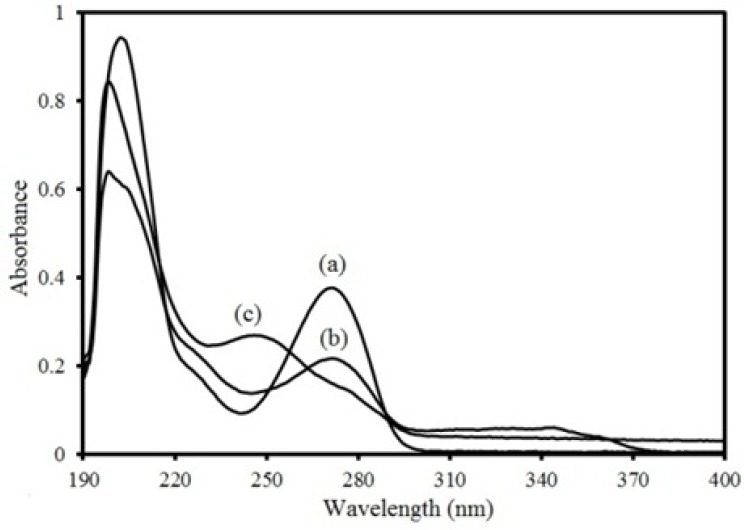
Electronic absorbance spectra of 6 ppm three compounds in methanol: (a) theophylline, (b) montelukast and (c) loratadine.

**Figure 3:**
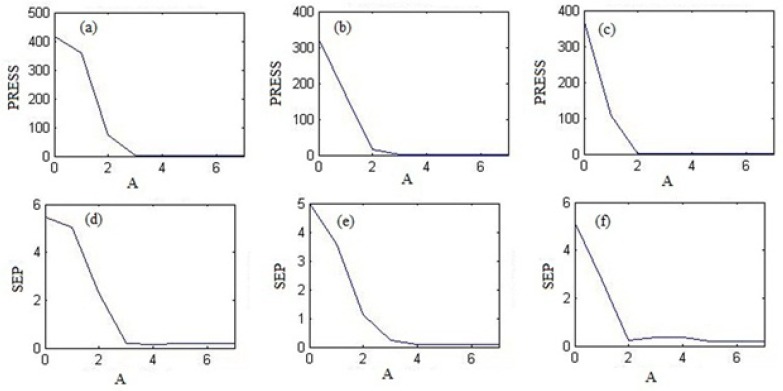
Variation of the PRESS as a function of the number of latent variables (A) (a) with PCR for theophylline, (b) with PLS1 for montelukast and (c) with HLA for loratadine. Plot of SEP vs. A (d) for theophylline by PCR regression, (e) for montelukast with PLS1 and (f) for loratadine with HLA.

*Artificial and unknown samples*

In order to apply the MVC1 methods, following manner was applied for artificial and unknown samples. Artificial samples were also prepared by mixing appropriate volumes of three stock solutions of theophylline, montelukast and loratadine in 10 mL volumetric flask and were diluted by methanol. Ten tablets of each drug were powdered in a mortar, separately. Then, a weight equivalent of one tablet of each drug was dissolved in 100 mL of methanol. After 40 min of stirring and 20 min of standing in the dark, a 10 mL of human plasma were spiked with 90 mL of different concentration levels of each drug. After well mixing, 4 mL of these solutions transferred into tubes and was centrifuged for 5 min at 10000 rpm to be sure of complete deproteinization of the plasma. The clear supernatant solution was withdrawn and transferred into a series of 10 mL volumetric flask and the flasks were completed by various volumes of methanol/plasma (90/10) to obtain a series of different concentration of these solutions. In otherwise, the blank solution (i.e. methanol/plasma, 90/10) was carried out according to the above process. These solutions then analyzed with Uv-Vis spectrophotometer to achieve the real analyzed data to apply PLS1, PCR and HLA methods.

**Table 1:** The concentration of three materials in the calibration set based on central composite design. Concentration values are expressed as mgL^−^^1^.

Analyte	1	2	3	4	5	6	7	8	9	10	11	12	13	14	15
THEO	14	14	14	2	2	2	14	2	8	8	14	8	8	2	8
MKST	14	14	2	14	2	14	2	2	8	8	8	14	2	8	8
LORA	14	2	14	14	14	2	2	2	8	2	8	8	8	8	14

**Table 2:** Prediction set composition and predicted values for theophylline (THEO) by PLS1, PCR and HLA regression. Concentration values are expressed as mgL^−1^.

**PLS1**		**PCR**		**HLA**	**PLS1**		**PCR**
**Sample No.**	**Exp.**	**Pred.**	**RE**	**Pred.**	**RE**	**Pred.**	**RE**
Test 1	11.00	10.54	-4.18	10.53	-4.27	10.56	-4.00
Test 2	11.00	10.72	-2.54	10.72	-2.54	10.72	-2.54
Test 3	11.00	10.82	-1.64	10.83	-1.54	10.84	-1.45
Test 4	3.00	2.97	-1.00	2.98	-0.67	2.93	-2.33
Test 5	3.00	2.98	-0.67	2.99	-0.33	2.93	-2.33
Test 6	3.00	2.87	-4.33	2.87	-4.33	2.83	-5.67
Test 7	11.00	10.86	1.27	10.87	-1.18	10.93	-0.66
Test 8	3.00	3.03	1.00	3.04	1.33	3.06	2.00
Test 9	7.00	7.14	2.00	7.14	2.00	7.13	1.86
Test 10	7.00	7.02	0.28	7.01	0.14	7.13	1.86
Test 11	11.00	11.01	0.09	11.01	0.09	11.08	0.73
Test 12	7.00	6.91	-1.28	6.90	1.43	6.94	0.86
Test 13	7.00	7.20	2.85	7.20	2.86	7.25	3.57
Test 14	3.00	3.21	7.00	3.18	6.00	3.14	4.67
Test 15	7.00	7.01	0.14	6.98	-0.28	6.58	6.00
$\overset{\text{̅}}{\mathrm{R.E.}}$ ^a^			2.02		1.93		2.70

a
$\overset{\text{̅}}{\mathrm{R.E.}}$ is the mean of relative error percentage.

**Table 3:** Prediction set composition and predicted values for montelukast (MKST) by PLS1, PCR and HLA regression. Concentration values are expressed as mgL^−1^.

		**PLS1**		**PCR**		**HLA**	
**Sample No.**	**Exp. **	**Pred. **	**RE**	**Pred. **	**RE**	**Pred. **	**RE**
Test 1	11.00	10.98	-0.18	11.06	0.54	10.94	-0.54
Test 2	11.00	11.03	0.27	11.06	0.54	10.95	-0.45
Test 3	3.00	3.09	3.00	3.11	3.67	3.06	2.00
Test 4	11.00	10.87	-1.18	10.94	-0.54	10.82	-1.64
Test 5	3.00	2.85	-5.00	2.86	-4.67	2.81	-6.33
Test 6	11.00	10.69	-2.82	10.73	-2.09	10.67	-4.71
Test 7	3.00	3.05	1.67	3.03	1.00	3.02	0.67
Test 8	3.00	2.99	-0.33	2.97	-1.00	2.97	-1.00
Test 9	7.00	7.41	5.86	7.43	6.14	7.39	5.57
Test 10	7.00	6.92	1.14	6.95	-0.71	6.90	-1.43
Test 11	7.00	6.97	-0.43	7.00	0.00	6.91	-1.28
Test 12	11.00	11.14	1.27	11.21	1.91	11.11	1.00
Test 13	3.00	3.08	2.67	3.08	2.67	3.06	2.00
Test 14	7.00	7.34	4.86	7.40	5.71	7.31	4.43
Test 15	7.00	7.23	3.28	7.68	9.71	7.25	3.57
$\overset{\text{̅}}{\mathrm{R.E.}}$ ^a^			2.26		2.73		2.44

a
$\overset{\text{̅}}{\mathrm{R.E.}}$ is the mean of relative error percentage.

**Table 4:** Prediction set composition and predicted values for loratadine (LORA) by PLS1, PCR and HLA regression. Concentration values are expressed as mgL^−1^.

		**PLS1**		**PCR**		**HLA**	
**Sample No.**	**Exp. **	**Pred. **	**RE**	**Pred. **	**RE**	**Pred. **	**RE**
Test 1	11.00	11.44	4.00	11.45	4.09	11.39	3.54
Test 2	3.00	3.15	5.00	3.15	5.00	3.01	0.33
Test 3	11.00	11.22	2.00	11.22	2.00	11.19	1.73
Test 4	11.00	11.50	4.54	11.51	4.64	11.50	4.54
Test 5	11.00	11.37	3.36	11.37	3.36	11.35	3.18
Test 6	3.00	3.19	6.33	3.20	6.67	3.09	3.00
Test 7	3.00	2.94	2.00	2.94	2.00	2.90	3.33
Test 8	3.00	3.00	0.00	2.99	0.33	3.03	1.00
Test 9	7.00	7.37	5.28	7.37	5.28	7.28	4.00
Test 10	3.00	3.10	3.33	3.11	3.67	3.13	4.33
Test 11	7.00	7.17	2.43	7.17	2.43	7.05	0.71
Test 12	7.00	7.24	3.43	7.25	3.57	7.23	3.28
Test 13	7.00	7.26	3.71	7.26	3.71	7.25	3.57
Test 14	7.00	7.27	3.86	7.28	4.00	7.25	3.57
Test 15	11.00	11.36	3.27	11.41	3.73	11.17	1.54
$\overset{\text{̅}}{\mathrm{R.E.}}$ ^a^			3.50		3.63		2.78

a
$\overset{\text{̅}}{\mathrm{R.E.}}$ is the mean of relative error percentage.

**Table 5:** Optimum number of factors and statistical parameters for calibration and prediction sets for three drugs.

		**THEO**			**MKST**			**LORA**	
**Parameters**	**PLS1**	**PCR**	**HLA**	**PLS1**	**PCR**	**HLA**	**PLS1**	**PCR**	**HLA**
A^a^	4	4	13	5	5	7	4	4	6
R^2^_CV_	0.998	0.998	0.998	0.999	0.999	0.999	0.998	0.998	0.998
SEP_CV_	0.173	0.170	0.174	0.081	0.091	0.085	0.173	0.195	0.174
R^2^_PRED_ RMSEP REP SEP_PRED_	0.996 0.184 2.627 0.177	0.997 0.173 2.476 0.167	0.995 0.214 3.000 0.207	0.994 0.187 2.674 0.180	0.990 0.264 3.774 0.255	0.993 0.195 2.787 0.188	0.991 0.292 4.173 0.282	0.990 0.301 4.300 0.290	0.993 0.251 3.590 0.251

a A is the number of factor or latent variable and obtained at minimum prediction residual error sum of squares (PRESS)^56^.

$\mathrm{PRESS}=\sum_{i=1}^{n}{\left(C_{\mathrm{pred}}-C_{\mathrm{act}}\right)}^{2}$

$\mathrm{RMSE}\left(\mathrm{CV or P}\right)={\left[\frac{1}{m-1}\sum_{1}^{m}{\left(C_{\mathrm{act}}-C_{\mathrm{pred}}\right)}^{2}\right]}^{1/2}\mathrm{REP}\left(\%\right)=\frac{100}{\overset{\text{̅}}{C}}{\left[\frac{1}{m-1}\sum_{1}^{m}{\left(C_{\mathrm{act}}-C_{\mathrm{pred}}\right)}^{2}\right]}^{1/2}$

$\mathrm{SEP}={\left[\frac{\sum_{i=1}^{m}{\left(C_{\mathrm{pred}}-C_{\mathrm{act}}\right)}^{2}}{m}\right]}^{2}R^{2}=1-\frac{\sum_{1}^{m}{\left(C_{\mathrm{act}}-C_{\mathrm{pred}}\right)}^{2}}{\sum_{1}^{m}{\left(C_{\mathrm{act}}-\overset{\text{̅}}{C}\right)}^{2}}$

C_pred_ is predicted concentration, C_act_ is the actual concentration of analyte, $\overset{\text{̅}}{C}$ is mean of real concentration in the prediction set and m is the number of samples in the prediction set (55, 58).

**Table 6:** Analytical figures of merit of the spectrophotometric method and PLS1, PCR and HLA regressions.

		**THEO**			**MKST**			**LORA**	
**Parameters**	**PLS1**	**PCR**	**HLA**	**PLS1**	**PCR**	**HLA**	**PLS1**	**PCR**	**HLA**
SEN	0.119	0.127	0.018	0.062	0.069	0.040	0.081	0.083	0.022
SEL	0.464	0.498	0.072	0.178	0.198	0.114	0.342	0.350	0.094
LOD	0.03	0.03	0.18	0.06	0.05	0.09	0.05	0.05	0.10
γ^ −1^	0.010	0.010	0.059	0.020	0.016	0.030	0.016	0.016	0.033

The unit of SEN is (AU. L.mg^–1^) that AU is absorbance unit, unit of γ (L mg^–1^) and unit of γ^–1^ and LOD (mg L^–1^).

**Table 7:** The comparison of LOD (mg L^-1^) of PCR regression described in this work with previously published work.

**Sample**	**This work**			**LOD (Ref.)**				
THEO	0.03	0.36 (14)	0.3 (12)	0.18 (9)	0.072 (15)	0.067 (16)	0.03 (18)	0.01 (4)
MKST	0.05	0.293 (21)	0.2 (28)	0.1 (25)	0.075 (29)	0.009 (26)	_	_
LORA	0.05	0.16 (30)	0.048 (39)	5.0×10^-4 ^(36)	2.5×10^-4 ^(33)	1.2×10^-5^ (38)	_	_

**Table 8:** Prediction result on artificial sample and commercial samples obtained with PLS1, PCR and HLA methods for theophylline. Concentration values are expressed as mgL^−1^.

			**Predicted**	
**Sample No.**	**Actual**	**PLS1**	**PCR**	**HLA**
Art 1^a^	3.00	2.99±0.09 (99.67) ^b^	2.99±0.07 (99.67)	3.07±0.06 (102.33)
Art 2	4.00	4.14±0.13 (103.50)	4.05±0.08 (101.25)	4.14±0.08 (103.50)
Art 3	6.00	6.02±0.06 (100.33)	6.01±0.05 (100.16)	6.04±0.05 (100.67)
Art 4	8.00	8.07±0.08 (100.87)	8.02±0.06 (100.25)	7.95±0.07 (99.37)
Art 5	9.00	9.06±0.09 (100.67)	9.05±0.08 (100.55)	9.13± 0.09 (101.44)
Art 6	0.00	–0.05±0.06 (–)	−0.05±0.05 (–)	−0.04±0.05 (–)
Art 7	8.00	8.12±0.10 (101.50)	8.07±0.09 (100.87)	7.92±0.08 (99.00)
Art 8 Unk 1^a^ Unk 2 Unk 3 Unk 4 Unk 5	0.00 10.00 12.00 13.00 12.00 13.00	–0.06±0.06 (–) 9.73±0.08 (97.30) 12.28±0.09 (102.33) 13.17±0.09 (102.57) 12.33±0.10 (102.75) 13.44±0.12 (103.38)	−0.07± 0.06 (−) 10.30±0.06 (103.00) 12.25±0.12 (102.08) 13.15±0.13 (101.15) 12.31±0.10 (102.58) 13.39±0.11 (103.00)	−0.05±0.07 (–) 10.38±0.12 (103.80) 12.33±0.11 (102.75) 13.23±0.10 (101.76) 12.42±0.09 (103.50) 13.47±0.13 (103.61)
	$\overset{\text{̅}}{x}$	101.33	101.32	101.97

a Art is artificial samples and Unk is unknown samples.

b The reported values of standard deviations (±S.D.) are obtained from four replicates. Recovery percentages are shown in parentheses.

**Table 9:** Prediction result on artificial sample and commercial samples obtained with PLS1, PCR and HLA methods for montelukast. Concentration values are expressed as mgL^−1^.

			**Predicted**	
**Sample No.**	**Actual**	**PLS1**	**PCR**	**HLA**
Art 1^a^	4.00	4.06±0.07 (101.50) ^b^	4.06±0.07 (101.50)	4.12±0.11 (103.00)
Art 2	5.00	5.02±0.17 (100.40)	5.15±0.17 (103.00)	5.03±0.14 (100.60)
Art 3	7.00	6.90±0.09 (98.57)	6.92±0.08 (98.86)	6.85±0.13 (97.85)
Art 4	10.00	9.97±0.10 (99.70)	9.85±0.14 (98.50)	10.00±0.10 (100.00)
Art 5	5.00	5.18±0.12 (103.60)	5.20±0.19 (103.80)	4.86±0.16 (97.20)
Art 6	10.00	10.16±0.12 (101.60)	10.16±.012 (101.60)	10.14±0.12 (101.40)
Art 7	0.00	–0.02±0.03 (–)	−0.02±0.04 (−)	−0.03±0.12 (–)
Art 8 Unk 1^a^ Unk 2 Unk 3 Unk 4 Unk 5	0.00 0.00 9.00 11.00 13.00 11.00	–0.03±0.03 (–) –0.32±0.13 (–) 8.75±011 (97.22) 10.63±0.09 (96.64) 12.73±0.07 (97.92) 11.27±0.12 (102.45)	−0.01± 0.04 (−) −0.31±0.13 (−) 9.34±0.13 (103.78) 10.51±0.08 (95.54) 12.61±0.07 (105.08) 11.26±0.10 (102.36)	−0.05±0.13 (–) −0.46±0.20 (–) 8.52±0.17 (94.66) 10.53±0.12 (95.73) 13.56±0.11 (104.31) 10.71±0.13 (97.36)
	$\overset{\text{̅}}{x}$	99.96	101.40	99.21

a Art is artificial samples and Unk is unknown samples.

b The reported values of standard deviations (±S.D.) are obtained from four replicates. Recovery percentages are shown in parentheses.

**Table 10:** Prediction result on artificial sample and commercial samples obtained with PLS1, PCR and HLA methods for loratadine. Concentration values are expressed as mgL^−1^.

			**Predicted**	
**Sample No.**	**Actual**	**PLS1**	**PCR**	**HLA**
Art 1^a^	4.00	4.08±0.13 (102.00) ^b^	4.08±0.13 (102.00)	4.06±0.21 (101.50)
Art 2	5.00	5.13±0.14 (102.60)	5.17±0.13 (103.40)	5.10±0.22 (102.00)
Art 3	9.00	8.96±.012 (99.56)	9.10±0.09 (101.11)	8.72±0.16 (96.89)
Art 4	10.00	10.08±0.14 (100.80)	10.08±0.14 (100.80)	10.02±0.21 (100.20)
Art 5	13.00	12.85±0.10 (98.84)	12.85±0.10 (98.84)	13.05±0.22 (100.38)
Art 6	10.00	10.29±0.09 (102.90)	10.28±.012 (102.80)	9.95±0.17 (99.50)
Art 7	4.00	4.02±0.14 (100.50)	4.12±0.11 (103.00)	4.00±0.13 (100.00)
Art 8 Unk 1 ^a^ Unk 2 Unk 3 Unk 4 Unk 5	0.00 0.00 12.00 14.00 12.00 14.00	–0.12±0.10 (–) –0.44±0.11 (–) 12.34±0.09 (102.83) 14.53±013 (103.78) 12.50±0.08 (104.17) 14.29±0.09 (102.07)	−0.17±0.15 (−) −0.47±0.12 (−) 12.32±0.09 (102.67) 14.48±0.14 (103.42) 12.53±0.07 (104.42) 14.28±0.10 (101.86)	−0.05±0.13 (–) −0.38±0.20 (–) 12.21±0.24 (101.75) 13.72±0.23 (98.00) 12.57±0.23 (104.75) 14.40±0.21 (102.86)
	$\overset{\text{̅}}{x}$	101.82	102.12	100.71

a Art is artificial samples and Unk is unknown samples.

b The reported values of standard deviations (±S.D.) are obtained from four replicates. Recovery percentages are shown in parentheses.

## Results and Discussion

*Electronic absorption spectra*

The electronic absorption spectra of THEO, MKST and LORA with concentration of 6 ppm are shown in Figure 2. As can be seen, the spectrum of each drug is overlapped with each other. Therefore, these compounds cannot be determined in the presence of each other by a singlevariate calibration procedure without prior separation. The method of multivariate calibration can be applied for determination of each drug in the mixtures. The composition data of the calibration sets are presented in Table 1. Due to overload of absorption value at λ_max_ 201 nm in mixture of compounds and low order of LDR, spectra were recorded in the region between 225 and 390 nm with 1.0 nm steps (165 points per spectrum). The same way was performed for samples in validation, artificial and unknown sets.

*Calibration and validation result*

Multivariate calibration methods demand a suitable experimental design of the standards belonging to the calibration set in order to have good predictions. The PLS1, PCR and HLA models were constructed for calibration data set that planned according CCD design (see Table 1.). Table 2. is shown data of prediction set composition, predicted values and relative error for THEO. As can be seen in this table, the minimum mean values of relative errors are 2.02, 1.93 and 2.70 for THEO with PLS1, PCR and HLA, respectively. This result indicated that minimum mean relative error for THEO was obtained by PCR model. Data of prediction set composition, predicted values and mean relative error for MKST and LORA with PLS1, PCR and HLA are presented in Tables 3. and 4, respectively. These tables indicate that the minimum mean values of relative errors are 2.26, 2.73 and 2.44 for MKST and 3.50, 3.63 and 2.78 for LORA with PLS1, PCR and HLA, respectively. Therefore, minimum mean relative error for MKST and LORA were obtained by PLS1 and HLA model, respectively.

Optimum number of factors (A), R^2^ and SEP that extracted form cross-validation (CV) and R^2^, RMSEP, REP and SEP for prediction set are listed in Table 5. This Table indicates that the best correlation coefficient value for prediction (R^2^_pred_) is 0.997 for THEO with PCR, 0.994 for MKST with PLS1 and 0.993 for LORA with HLA models. This also specifies that minimum value of REP is 2.476 mg L^–1^ for THEO with PCR, 2.674 mg L^–1^ for MKST with PLS1 and 3.590 mg L^–1^ for LORA with HLA model. Consequently, it can be concluded that all of R^2^_pred_ values are in agreement with REP for all drugs. As can be seen in Table 2, 3 and 4. minimum mean relative error are obtained with PCR, PLS1 and HLA model for THEO, MKST and LORA, respectively that confirm result of Table 5.

Plots of PRESS and SEP versus number of factor (A) were obtained from PLS1, PCR and HLA models for all drugs. As an example, Figure 3. shows these plots for THEO by PCR regression, for MKST by PLS1 regression and for LORA with HLA regression. As can be seen in this Figure, the optimum number of factors for prediction of THEO, MKST and LORA concentrations in prediction set and unknown samples were obtained 4, 5 and 6 with PCR, PLS1 and HLA regressions. The optimum numbers of factors for these drugs by other models are prsented in Table 5.

*Analytical figures of merit*

Determination of figures of merit (FOM) is an important necessary for the validation of chemometric methods. FOM, such as selectivity (SEL), sensitivity (SEN), analytical sensitivity (*γ*) and limit of detection (LOD), can be predicted and utilized to compare analytical methods. When expressing FOM for multivariate calibration methods, the portion of the signal that relates to the analyte is more important than the total signal.

This unique signal is called net analyte signal and is defined as the part of the signal that is orthogonal to the signal of the interferences present in the sample. The SEL is a measure, ranging from 0 to 1, of how unique the spectrum of the analyte is compared with the other species. It specifies that the part of the total signal that is not lost due to spectral overlap, and can be defined in the multivariate context by resorting to NAS calculation (57):

$\mathrm{SEL}=\frac{\left\|\left.s_{k}^{*}\right\|\right.}{\left\|\left.s_{k}\right\|\right.}$

(1)

where || || means the Euclidian norm of vector and *s*_k_ is a spectrum containing analyte *k *at unit concentration and is its corresponding NAS (59). The sensitivity measures the changes in response as a function of the concentration of a particular analyte (57), and is stated by the following Eq.:

$\mathrm{SEN}=\left\|\left.s_{k}^{*}\right\|\right.$

(2)

A more informative FOM is the analytical sensitivity (*γ*), which is defined, in analogy with univariate calibration, as the ratio between SEN and the instrumental noise (*ε*), according to Eq. 3:

$\gamma =\frac{\mathrm{SEN}}{\left\|\left.\varepsilon \right\|\right.}$

(3)

where ||*ε*|| is a measure of the instrumental noise. The value of ||*ε*|| may be estimated from the standard deviation in the NAS of several blanks. With the inverse of *γ *(*γ*^-1^), it is possible to establish a minimum concentration difference that is marked by the analytical method in the absence of experimental error. Concerning the limit of determination, the following simple equation has been proposed for its estimation (60, 61):

$\mathrm{LOD}=\frac{3\left\|\left.\varepsilon \right\|\right.}{\left\|\left.s_{k}^{*}\right\|\right.}$

(4)

Estimated FOM for THEO, MKST and LORA were determined with the PLS1, PCR and HLA models and were shown in Table 6. According to data of this table, the reverse of analytical sensitivity (*γ*^-1^) value is small for MKST and LORA by PCR model and for THEO by PLS1 model that suggest these models are satisfactory models for this multivariate analysis. Results also specify that the selectivity is higher for THEO (0.498), LORA (0.350) and MKST (0.198) with PCR model. Finally, LOD is better (lower value) for THEO, LORA with PLS1 and PCR models meanwhile PCR model is suitable for MKST.

The LOD of MVC1 method is compared with the reported value in the previous work for three drugs and presented in Table 7. (4, 9, 12, 14–16, 18, 21, 25, 26, 28–30, 33, 36, 38, 39). As can be seen in this Table, the PCR method showed somewhat low LOD against previously reported works for THEO (9, 12, 14–16), for MKST (21, 25, 28, 29) and for LORA (30, 39).

*Analysis of artificial and unknown samples*

Table 8. are presented predicted values and recovery percentage by PLS1, PCR and HLA models for THEO in artificial and unknown samples. This table indicates that the mean recovery percentage for THEO is 101.33, 101.32 and 101.97 with PLS1, PCR and HLA regressions, respectively. Results indicated that the best calibration model for direct determination of THEO in the commercial sample containing MKST and LORA is PCR regression. It can be stated that this result is in agreement with achieved results in Table 2. for calculation of THEO concentration in prediction set.

The similar data for determination of MKST in artificial and unknown samples are listed in Table 9. According to this table, the mean recovery obtained 99.96, 101.40 and 99.21 for MKST using PLS1, PCR and HLA regressions, respectively. Therefore, the best calibration model for direct determination of MKST in the present of THEO and LORA in the artificial and unknown samples is PLS1 regression. It can be noted that this result is in agreement with obtained results in Table 3. for calculation of MKST concentration in prediction set.

The same data for determination of LORA in artificial and unknown samples are presented in Table 10. Table 10. shows that the mean recovery obtained 101.82, 102.12, and 100.71 for LORA with PLS1, PCR and HLA regressions, respectively. As a result, the best calibration model for direct determination of LORA in the present of THEO and MKST in the artificial and unknown samples is HLA regression. This result is in agreement with obtained results in Table 4. for calculation of LORA concentration in prediction set.

## Conclusion

Simultaneous determination of theophylline (THEO), montelukast (MKST) and loratadine (LORA) was achieved by PLS1, PCR and HLA using data extracted from UV–Vis spectrum of above drugs. These methods were advanced for the determination of THEO, MKST and LORA in the artificial and pharmaceutical tablet samples in human plasma. The high recovery values for artificial and commercial samples indicate good accuracy of the methods for all drugs. Results obtained from this study specified that the best model for THEO in the presence of MKST and LORA is PCR regression, for MKST in the presence of THEO and LORA is PLS1 regression and for LORA in the presence of THEO and MKST is HLA regression. Compared to other procedures techniques, the proposed method which is characterized by minimum sample pretreatment provides a fast, accurate and convenient alternative for the simultaneous determination of above compounds in routine quality control of their pharmaceutical formulations. These methods were found selective for THEO, MKST and LORA in the presence of common excipients, dyes and coating materials in the sugar-coated tablet formulation analyzed.
